# Notes on the Use of Koch’s Lymph1Translated for the Buffalo Medical and Surgical Journal, by Dr. Julius Pohlman.

**Published:** 1891-05

**Authors:** E. Thorner

**Affiliations:** 15 Anhalt Street; Berlin


### NOTES ON THE USE OF KOCH'S LYMPH.[1]

Special Contribution

By DR. E. THORNER, Berlin.

All records published up to date of cases treated with the lymph are necessarily incomplete, because they have been under observation only a short time. In view of the many serious objections raised against the use of tuberculine, it behooves us to decide whether it is a desirable agent in the hands of the general practitioner. The question assumes a greater importance, because the lymph can now be obtained quite readily, and the former excuse that it could not be purchased, is no longer of value.

The objections raised against the use of the tuberculine can conveniently be classed under the following heads:

1. It can produce a too powerful reaction, the fever following an injection can be of a very high degree, and the treatment can even be followed by collapse.

1. Translated for the Buffalo Medical and Surgical Journal, by Dr. Julius Pohlman.

2. Tuberculine can produce hyperemia in different organs; hemorrhage in the lungs; in the brain serious disturbances and coma.

3. The local swellings formed with the reaction around tubercular nodules can become dangerous if situated, for instance, in the larynx or trachea.

4. Nephritis has been caused by the lymph, and in albuminuria an increased excretion of albumin has been observed.

5. Pneumonia of a peculiar form, pleurisis, as well as localized inflammations of the lungs have been observed to follow the injection. But the most serious objection made is that through it the bacilli are driven into the general circulation, producing miliar-tuberculosis.

6. And, lastly, it is claimed that tuberculine is unreliable, its action uncertain, especially in old and in quite recent cases.

Against these objections we can bring the long series of brilliant results obtained by Dr. Koch, himself, and verified by a long list of careful, unprejudiced observers. Personally, I have had the lymph since November 20, 1890, and, although I have used it in a large variety of forms of tuberculosis, I have had only one fatal case,— a boy, *æt.* fourteen, who suffered from lung, laryngeal, and intestinal tuberculosis, and was already in an advanced stage of fever when he came under my treatment in the first week of December. The injections were given with the greatest care, beginning with 0.5 mg., and increasing slowly. The fever remained, but the general health, as well as the lung and throat symptoms, improved somewhat; the intestinal trouble, however, grew worse, the diarrhea increased and could not be stopped, and, as I was afraid of a perforation of the intestines, the injections were discontinued on January 8th. At the end of January the boy died, but the tuberculine has been beneficial in every other case treated by me; even cases with large cavities in the lungs improved. A gentleman, *æt.* thirty-eight, who had tried all forms of treatment at the different health resorts, had an enormous cavity in one lung, but no fever, when he came to me. Under the influence of the lymph the bacilli in the sputa decreased as well as the quantity of the sputum itself, and the patient, up to date, has gained seven pounds in weight. The cavity in the lung remains, but it can be assumed that it is clean today. Two other cases of severe lung trouble have improved considerably, although it would be folly to talk about a cure after so short a period of observation. Several cases treated in the earliest stages of lung tuberculosis show no symptoms of the disease, whatever, today; of course all these favorable results cannot overthrow the objections raised, and we must consider seriously what care has to be exercised by the general practitioner in order to avoid dangerous consequences.

Before Dr. Koch published the formula of the lymph, the wildest speculations as to its action were frequent. Today, we know that the glycerine extract contains only one per cent. of the active principle. If, therefore, a patient weighing fifty *kg.* receives an injection of 1 *cg.* of the tuberculine and a reaction occurs, then the result is obtained by a quantity which stands in proportion to the body weight as one to five hundred millions. There are not many substances which will act in such minute quantities.

We can claim for tuberculine that it produces an increased activity in the tubercular lesions. The living bacilli excrete a substance which slowly produces a necrosis of the surrounding tissues. We add to this the action of the injected tuberculine and the combined activities are sufficient to form a coagulation necrosis around the nodule, and a line of demarcation between the healthy and diseased tissue, which prevents a farther penetration of the bacilli into the sound surroundings. The cheesy degeneration, which we have considered as an unfavorable symptom, when viewed from this new standpoint, becomes a protective measure of the body against further invasion of the bacilli. When left to itself, the necrosis is not sufficiently a barrier and the bacilli can penetrate it and enter healthy tissues, but when we add to the excretion of the bacilli itself the tuberculine, the dividing line is formed rapidly and the bacilli die or are eliminated, as the case may be.

Hopes were entertained at first that tuberculine would ensure immunity to the body against attacks of the bacillus tuberculosis, but over 1,000 experiments made on animals seem to settle the question; and, although our hopes for immunity have not been realized, we know for certain that the animals are safe against any infection by the bacilli as long as they are under treatment by tuberculine; and right here it is well to notice that animals can bear a dose from 1,500 to 3,000 times as large as man.

From a therapeutical standpoint we have, at least, a large variety of important remedies with which to combat the unfavorable symptoms that may arise from the use of tuberculine. The severity of the reaction fever can be undoubtedly lessened when we use very minute doses. The initial injection should be one *mg.* for each fifty *kg.* of body weight and increased slowly. If the patient loses flesh during the early stages of the treatment, we can interrupt it for one or two weeks and begin again with very small doses. As a rule, the patient bears the second series of injections better than he did the first, and a too strong reaction is thereby avoided. To guard against collapse any symptoms of heart disease or organic lesions, as well as extreme youth and old age, should make us very careful in the use of tuberculine. A possible brain hyperemia prohibits the use of the remedy in cases ot tubercular basilar-meningitis, unless the prognosis is more favorable after trephining. As the lungs can become hyperemic from an injection, its use cannot be recommended after a recent hemorrhage, but some time and a complete rest of the patient is necessary before beginning the treatment.

One of my patients who suffered for many weeks from lung hemorrhage, has improved very materially since the use of the lymph, and has not had any attack now for ten weeks. The initial hemorrhage, which is so often the first symptom of lung tuberculosis, never prevents me from using the tuberculine after a certain lapse of time for the patient to rest.

Severe swelling of trachea and larynx are, of course, more dangerous in cases of lupus than in the ordinary forms of tuberculosis, and in such cases the physician should always be prepared to perform tracheotomy ; hence Koch's method is inadvisable in country practice where patient and physician live a long distance apart. The localized inflammations observed in the lungs after the injection are beyond doubt swellings around tubercular nodules and disappear, as a rule, in the course of the treatment.

Pneumonia, due to the tuberculine, becomes dangerous on account of the serverity of the fever and the reduction of air surface in the lungs. Hence, its use is contra-indicated in cases of pleuritic exudates, pneumothorax and anywhere where the air capacity has been materially reduced from one cause or another ; in such cases the existing troubles must be cured first before beginning the injection of tuberculine. In the same way an existing nephritis contra-indicates the use of the remedy, because the increased tissue change in the body requires healthy kidneys for its elimination.

The most serious, however, of all objections raised, is that the tuberculine liberates living bacilli, throws them into the general circulation, and thereby produces a general in the place of a localized tuberculosis, and that the so formed general tuberculosis is not affected by the tuberculine.

I do not deny the possibility of such occurrences, but doubt the latter inference, because in experiments made on animals such a general tuberculosis was amenable to treatment by tuberculine, just as well as other forms.

That the remedy does not influence very old or very recent tubercular nodules may be accounted for by the varying conditions of the circulation; in the former it can prevent the access of the tuberculine to the nodule, while in the latter the bacilli have not excreted a sufficient quantity of substance to permit a combined action of tuberculine and excretion; we can assume, however, that repeated injections will attack these most recent nodules just as well.

When we now place all the objections to Koch's method in one scale and the discouraging prognosis of tuberculosis in the other, we must admit that in tuberculine we have found a remedy which *can* work injury, but the application of which is *most* beneficial and almost harmless *if applied early enough;* for when the majority of the complications have not yet appeared, the fever is either very light or totally absent, and small tubercular nodules can be eliminated with ease.

Of course the early diagnosis of tuberculosis is most important, and the possession of a good microscope and well trained capacity to use it are absolutely necessary. The mere name of "bacteriological microscope" or "oil immersion," is not sufficient; such instruments should have been tested by experts to demonstrate their usefulness. Taking these points for granted, the general practitioner will be often called upon to diagnosticate tuberculosis in its early stages, because as family physician he knows the patient, the family history, and can often determine the presence of the disease long before the patient thinks himself sick. The physician, however, who can diagnosticate cases of recent tuberculosis must also be in the position to treat and cure his patient with the most modern remedies. Certainly a patient would rather allow his family physician to treat him with the tuberculine than place himself under the care of a stranger or go to a hospital. Not until every general practitioner knows how and when to use the tuberculine, will the hopes be realized which its famous discoverer expressed, when he presented this unique remedy to the world of science.

[Since Dr. Thorner wrote the above, he tells me that all his patients, up to date, are doing well under the treatment, and that some of them have increased as much as twenty pounds in weight; but he strongly advises to continue the treatment not only until all the bacilli have disappeared from the sputum, but until repeated injections of guinea pigs with the sputum fail to produce any tubercular lesions in these animals.—TRANSLATOR.]

15 ANHALT STREET, APRIL 3, 1891.

